# Genome Sequence of a Classical Swine Fever Virus of Subgenotype 2.1, Isolated from a Pig in Japan in 2018

**DOI:** 10.1128/MRA.01362-18

**Published:** 2019-01-17

**Authors:** Tatsuya Nishi, Ken-ichiro Kameyama, Tomoko Kato, Katsuhiko Fukai

**Affiliations:** a Exotic Disease Research Unit, Division of Transboundary Animal Diseases, National Institute of Animal Health, National Agriculture and Food Research Organization, Kodaira, Tokyo, Japan; University of California, Riverside

## Abstract

In 2018, classical swine fever virus (CSFV) was detected in Japan. Here, we report the whole-genome sequence of CSFV/JPN/1/2018. This virus is closely related to isolates in East Asia and is classified under subgenotype 2.1. This is the first detection of a CSFV of this lineage in Japan.

## Abstract (precis)

In 2018, classical swine fever virus (CSFV) was detected in Japan. Here, we report the whole-genome sequence of CSFV/JPN/1/2018.

## ANNOUNCEMENT

Classical swine fever virus (CSFV) belongs to the Pestivirus genus within the Flaviviridae family. Its genome is composed of a single-stranded, positive-sense RNA approximately 12.3 kb long that contains a large open reading frame (ORF) encoding a polyprotein of 3,898 amino acids, with a 5′ untranslated region (UTR) and a 3′ UTR at either end (1). CSFV isolates are divided into three genotypes (1, 2, and 3) and 10 subgenotypes (1.1 to 1.3, 2.1 to 2.3, and 3.1 to 3.4) (2, 3). Subgenotype 2.1 isolates have been further divided into 2.1a, 2.1b, and 2.1c, and subgenotype 2.1d was confirmed in 2014 (4 – 6). In East and Southeast Asia, classical swine fever (CSF) outbreaks are largely due to the circulation of several genotypes of CSFV isolates (7). Because CSFV is the least variable member within the highly variable genus Pestivirus (2), a region of high variability and adequate length is required for reliable phylogenetic analyses and molecular epidemiological investigations.

In this study, tonsil samples were collected from a pig in a pig farm in the central part of Japan that showed clinical symptoms of CSFV in September 2018. The sample was homogenized to make a 10% (wt/vol) suspension with Dulbecco’s modified Eagle’s medium and nutrient mixture F-12 (Life Technologies), and its viral RNA was extracted using the High Pure viral RNA kit (Roche Diagnostics). First-strand cDNA synthesis was performed using SuperScript III reverse transcriptase (Life Technologies) and the CSFV-specific primers 2R (5′-CTGCTGCGGCCCTCAAGGGGAT-3′) and 4R (5′-ACTATGGACGTCAGGATTTTCC-3′). The whole genome of the virus, which was approximately 12.1 kb, was amplified by PCR with PrimeStar Max DNA polymerase (TaKaRa) and four CSFV-specific primer sets, 1F (5′-ATGCCCWTAGTAGGACTAGCA-3′) and 1R (5′-CAATACTGGTTTGACCTGGA-3′), 2F (5′-ACTTTGGATTTGGGCTGTGCC-3′) and 2R, 3F (5′-GACTGACGAGTCCGAATACGG-3′) and 3R (5′-CGGGGTCTCCCCTGTGGTC-3′), and 4F (5′-GCGTATCTGGGTGAGAAACCTA-3′) and 4R. Libraries were prepared using the Ion Xpress Plus fragment library kit (Life Technologies) by fragmenting DNA for 8 min, generating fragments of 300 bp, on average, which were barcoded using Ion Xpress barcodes (Life Technologies). The libraries were then size selected by 2% E-Gel SizeSelect (Invitrogen). Fragment size and concentration were measured by an Agilent Bioanalyzer. Libraries were amplified using the Ion OneTouch 2 system with an Ion PGM template OT2 200 kit (Life Technologies) and sequenced on an Ion Torrent PGM sequencer using the 314 chip (version 2) and Ion PGM sequencing 200 kit (version 2) (Life Technologies). Using the Torrent suite software (version 5) with default parameters (Life Technologies), 39,355 reads in total (6,009,632 bp) were assembled and mapped to the reference genome of CSFV strain SDZC150601 (GenBank accession number MF150646) with a read depth of coverage of more than 10. The final assembly of CSFV/JPN/1/2018 was 12,094 nucleotides (nt) in length, with 98.4% genome coverage, 47.5% G+C content, and an average coverage depth of 488.8×. This sequence is the most closely related to CSFV isolate BJ2-2017 (GenBank accession number MG387218), which is classified into subgenotype 2.1d, with a 98.9% percent nucleotide identity and no insertions or deletions, based on NCBI BLAST analysis (analyzed 30 November 2018 at https://blast.ncbi.nlm.nih.gov/Blast.cgi). The genome was annotated using GENETYX software (version 12) with the CSFV strain BV-P (GenBank accession number DQ314582) as the reference sequence. The sequence of CSFV/JPN/1/2018 includes a 5′ UTR of 253 nt, a single ORF of 11,697 nt encoding a polyprotein of 3,898 amino acids, and a 3′ UTR of 144 nt.

In conclusion, CSFV/JPN/1/2018 was detected from a domestic pig following an outbreak in 2018 in Japan. Our findings confirm the first outbreak in Japan caused by CSFV subgenotype 2.1. CSFV subgenotype 2.1 has continued to spread into East and Southeast Asia, and therefore epidemiological information and monitoring of the virus is important for developing appropriate control strategies against this disease.

### Data availability.

The genome nucleotide sequence of CSFV/JPN/1/2018 has been deposited in GenBank (accession number LC425854). The raw sequence reads were deposited in the Sequence Read Archive under BioProject accession number PRJDB7640 and SRA project accession number DRP004569.

## References

[1] Meyers G, Thiel HJ 1996 Molecular characterization of pestiviruses. Adv Virus Res 47:53–118. doi:10.1016/S0065-3527(08)60734-4. 8895831

[2] Lowings P, Ibata G, Needham J, Paton D 1996 Classical swine fever virus diversity and evolution. J Gen Virol 77:1311–1321. doi:10.1099/0022-1317-77-6-1311. 8683221

[3] Paton DJ, McGoldrick A, Greiser-Wilke I, Parchariyanon S, Song JY, Liou PP, Stadejek T, Lowings JP, Björklund H, Belák S 2000 Genetic typing of classical swine fever virus. Vet Microbiol 73:137–157. doi:10.1016/S0378-1135(00)00141-3. 10785324

[4] Deng MC, Huang CC, Huang TS, Chang CY, Lin YJ, Chien MS, Jong MH 2005 Phylogenetic analysis of classical swine fever virus isolated from Taiwan. Vet Microbiol 106:187–193. doi:10.1016/j.vetmic.2004.12.014. 15778024

[5] Jiang DL, Gong WJ, Li RC, Liu GH, Hu YF, Ge M, Wang SQ, Yu XL, Tu C 2013 Phylogenetic analysis using E2 gene of classical swine fever virus reveals a new subgenotype in China. Infect Genet Evol 17:231–238. doi:10.1016/j.meegid.2013.04.004. 23608662

[6] Zhang H, Leng C, Feng L, Zhai H, Chen J, Liu C, Bai Y, Ye C, Peng J, An T, Kan Y, Cai X, Tian Z, Tong G 2015 A new subgenotype 2.1d isolates of classical swine fever virus in China, 2014. Infect Genet Evol 34:94–105. doi:10.1016/j.meegid.2015.05.031. 26031602

[7] Tu C, Lu Z, Li H, Yu X, Liu X, Li Y, Zhang H, Yin Z 2001 Phylogenetic comparison of classical swine fever virus in China. Virus Res 81:29–37. doi:10.1016/S0168-1702(01)00366-5. 11682122

